# Neuroprotective Strategies for Ischemic Stroke—Future Perspectives

**DOI:** 10.3390/ijms24054334

**Published:** 2023-02-22

**Authors:** Matteo Haupt, Stefan T. Gerner, Mathias Bähr, Thorsten R. Doeppner

**Affiliations:** 1 Department of Neurology, University of Göttingen Medical School, 37075 Göttingen, Germany; 2 Department of Neurology, University Hospital Giessen, 35392 Giessen, Germany; 3 Center for Mind, Brain and Behavior (CMBB), University of Marburg and Justus Liebig University Giessen, 35032 Marburg, Germany; 4 Department of Anatomy and Cell Biology, Medical University of Varna, 9002 Varna, Bulgaria; 5 Research Institute for Health Sciences and Technologies (SABITA), Medipol University Istanbul, 34810 Istanbul, Turkey

**Keywords:** cerebral ischemia, neuroprotection, stroke, stem cells, extracellular vesicle, microbiota–gut–brain axis

## Abstract

Ischemic stroke is the main cause of death and the most common cause of acquired physical disability worldwide. Recent demographic changes increase the relevance of stroke and its sequelae. The acute treatment for stroke is restricted to causative recanalization and restoration of cerebral blood flow, including both intravenous thrombolysis and mechanical thrombectomy. Still, only a limited number of patients are eligible for these time-sensitive treatments. Hence, new neuroprotective approaches are urgently needed. Neuroprotection is thus defined as an intervention resulting in the preservation, recovery, and/or regeneration of the nervous system by interfering with the ischemic-triggered stroke cascade. Despite numerous preclinical studies generating promising data for several neuroprotective agents, successful bench-to-bedside translations are still lacking. The present study provides an overview of current approaches in the research field of neuroprotective stroke treatment. Aside from “traditional” neuroprotective drugs focusing on inflammation, cell death, and excitotoxicity, stem-cell-based treatment methods are also considered. Furthermore, an overview of a prospective neuroprotective method using extracellular vesicles that are secreted from various stem cell sources, including neural stem cells and bone marrow stem cells, is also given. The review concludes with a short discussion on the microbiota–gut–brain axis that may serve as a potential target for future neuroprotective therapies.

## 1. Introduction

Stroke is the leading cause of death and the major cause of acquired physical disability globally, with a worldwide incidence of 13.7 million strokes in 2016 compared to 11.6 million in 2011 [1]. The demographic change, as well as the rising incidence of cardiovascular diseases, aggravates the aforementioned trend. The therapeutic options are still limited to causal recanalization therapies, including systemic thrombolysis and interventional thrombectomy. However, only a small number of stroke patients benefit from these treatments due to time constraints and restrictive selection criteria. As a result, adjuvant therapy is urgently required.

The development of neuroprotective techniques is one of the primary therapeutic research approaches. Neuroprotection is thereby defined as an effect resulting in the preservation, recovery, or regeneration of the nervous system, its cells, structure, and function by inhibiting the pathogenic cascade [2]. The pathogenic stroke cascade thereby includes two main phases. The first phase is characterized by acute injury and neuronal cell loss within minutes, hours, and days due to ischemia. The second phase includes neurodegenerative processes occurring days, weeks, and even months after the ischemic event. Modulating these phases could lead to acute neuroprotection, as well as long-term neuroregeneration.

To this date, hundreds of potential neuroprotective drugs have shown promising preclinical evidence. Unfortunately, none of them have been successfully transferred to daily clinical routines [3]. Developing neuroprotective strategies for future stroke therapy, however, still remains a large and promising field of research, with many research groups working in the field worldwide.

The present review will provide a brief summary of ischemic stroke pathogenesis, identifying prospective targets for neuroprotective treatments. An overview of selected neuroprotectants that have already been moved to clinical trials is also presented. Following that, potential therapeutics based on stem cells and extracellular vesicles will be discussed, and finally, the brain–gut axis as a new neuroprotective approach will be reviewed.

## 2. Ischemic Stroke Pathophysiology

The ischemic-triggered stroke cascade, including neuroinflammation, blood–brain barrier (BBB) opening, and finally, cell death, offers a variety of possible targets for neuroprotective strategies. A narrow review of the complex stroke pathogenesis is provided here.

Ischemic stroke is defined as an acute neurological deficit caused by an interruption of the blood supply to part of the brain. At the molecular level, the impaired cerebral blood perfusion results in acute oxygen and glucose deficiency, leading to reduced adenosine triphosphate (ATP) production, followed by lactate acidosis and disturbed cellular homeostasis [4]. Lactate acidosis causes cell damage by disrupting the brain’s normal acid-base balance. Further, ATP deficiency results in the failure of ATP-dependent ion transport pumps leading to cellular depolarization and opening of voltage-gated Ca^2+^-, Na^+^-, and K^+^-channels [4]. This is followed by a large influx of Ca^2+^ and Na^+^, as well as an efflux of K^+^, overall triggering the release of glutamate, resulting in glutamate-mediated extracellular excitotoxicity [5]. Importantly, the enhanced intracellular Na^+^ influx causes cytotoxic edema [5]. The enhanced intracellular Ca^2+^ concentration activates proteases and lipases, as well as leads to the release of free radicals, degrading essential cellular components such as mitochondria. Degraded mitochondria cause cell death by producing intrasaccular toxins and apoptotic proteins. Necrotic cells release glutamate and trigger an inflammatory response leading to further damage to surrounding cells. Parallel to excitotoxicity, hypoxia- and reperfusion-triggered neuroinflammation is an important factor in stroke pathophysiology. Shortly after hypoxia, resident microglia are activated by the neuronal release of damage-associated molecular patterns (DAMPs) [4]. These activated microglia move to the infarct core and penumbra, where they mediate harmful and/or protective effects depending on their subtype [6]. However, to date, there is no consensus about the exact role of microglia in ischemic stroke, keeping the topic still a subject of current research [6]. Some subtypes of activated microglia secrete pro-inflammatory cytokines and free radicals such as nitric oxide. Furthermore, hypoxia enhances the expression of transcriptional factors such as NF-κB, hypoxia-inducible factors, and interferon regulatory proteins, resulting in increased synthesis of pro-inflammatory effector proteins and amplifying neuroinflammation. Interleukins (IL), particularly IL-1, promote the expression of cell adhesion molecules such as intercellular adhesion molecule one and vascular cell adhesion molecule one on the surface of the BBB [4]. These cell adhesion molecules serve as adhesion ligands for migrating leukocytes. Invading neutrophils secrete matrix metalloproteinases, which lead to the breakdown of tight junctions that physiologically ensure the integrity of the BBB [7]. The loss of vascular integrity results in a breakdown of the BBB, yielding aggravated extravasation of immune cells into the brain parenchyma and the formation of brain edema [7]. The latter results in neuronal tissue loss by cell death, such as apoptosis, necrosis, or others [4].

## 3. Current Stroke Treatment

**Figure 1:**
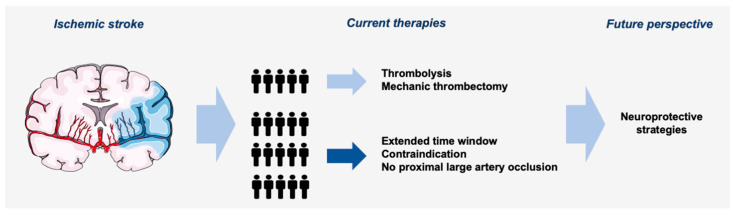
Current ischemic stroke treatment and its limitations.

Depending on contraindications, clinical criteria, and imaging findings, stroke patients may be eligible for treatment with intravenous thrombolysis, endovascular thrombectomy, or both. However, the application of these efficacious treatments is limited to a small percentage of patients due to strict selection criteria, including imaging characteristics and a narrow time window to start treatment. The following sections will go through therapy concepts, contraindications, and restrictions. Additionally, an overview is given in Figure 1.

### 3.1. Systemic Thrombolysis

Intravenous systemic thrombolysis with a fibrinolytic drug, primarily alteplase (rt-PA), is the standard of care for acute stroke treatment. Alteplase is a second-generation thrombolytic drug and the recombinant form of human tissue plasminogen activator. Mechanistically, alteplase converts plasminogen to the proteolytic enzyme plasmin, which lyses fibrinogen and fibrin. The fibrinolysis results in the breakdown of the blood clot and restore the blood flow to the brain.

The European Stroke Organization recommends intravenous thrombolysis (IVT) with alteplase for individuals with acute ischemic stroke presenting within 4.5 h after symptom onset [8]. If mechanical thrombectomy is not feasible, IVT may also be performed in stroke patients with known onset between 4.5 h and 9 h that have a CT or MRI core/perfusion mismatch [8]. Finally, patients with acute ischemic stroke on awakening from sleep, known as wake-up stroke, who were last seen well more than 4.5 h earlier showing an MRI DWI-FLAIR mismatch and for whom mechanical thrombectomy is either not indicated or not planned, should also receive intravenous thrombolysis with alteplase [8]. Importantly, extending the therapeutic window in patients who meet distinct clinical and imaging criteria is under discussion.

On-label thrombolysis therapy has several contraindications, such as recent intracranial hemorrhage, intracranial neoplasm, severe uncontrolled hypertension, and ischemic stroke within three months. Additionally, several existing relative contraindications, for example, severe hypertension at presentation (systolic blood pressure >180 mmHg or diastolic blood pressure >110 mmHg), pregnancy, dementia, and a history of stroke, should also be considered [9]. Complications of IVT with alteplase include symptomatic intracranial hemorrhage, major systemic hemorrhage, and angioedema. The occurrence of these complications varies between 2–6% [9].

### 3.2. Mechanical Thrombectomy

Mechanical thrombectomy is recommended for patients with acute ischemic stroke caused by large vessel occlusion of cerebral arteries. Patients with a National Institutes of Health stroke scale score of six or higher, who have undergone a non-contrast head CT, can be treated within 6 h from their last known well time [10]. In certain cases, the treatment time window may be extended up to 24 h, such as in patients who meet the criteria outlined in the DAWN and DEFUSE-3 trials and have evidence of a penumbra on perfusion CT or diffusion-weighted MRI scans [10]. Importantly, intraarterial mechanical thrombectomy is often combined with intravenous thrombolysis. Thereby, treatment with thrombolysis prior to thrombectomy is known as “bridging therapy” [11]. The catheterization for intraarterial mechanical thrombectomy is usually performed by femoral artery puncture. Thereafter, the catheter is guided to the internal carotid artery and to the site of the intracranial artery occlusion. Two methods, which could also be combined, are used to remove the occlusion, i.e., (I) aspiration devices and (II) stent retriever [11]. Catheter aspiration devices aspirate the thrombus from the occlusion side. A stent retriever, after reaching the thrombus through the catheter, is deployed into the thrombus and removes it as the device is pulled back. The approach chosen is partly determined by local expertise and availability.

### 3.3. Supportive Care

Supportive care in stroke therapies has the main goal of preventing secondary neurological damage and management of stroke-related complications. The blood pressure should be less than 180/105 mmHg within the first 24 h after thrombolysis. However, for patients without thrombolysis, reducing blood pressure lower than 220/120 mmHg has no benefit [9]. Medical care, including appropriated glycemic control, prevention of hyperthermia, prevention of deep vein thrombosis, antiplatelet treatment, and statin therapy in specific patient groups, improves outcomes. Early mobilization lowers the risk of complications and improves outcomes. Depending on the specific stroke etiology, surgery or intervention may be necessary to prevent further stroke, for example, in the case of severe symptomatic stenosis of the internal carotid artery.

### 3.4. Limitations

Recanalizing strategies are limited to narrow time windows and a specific imaging profile of stroke (see above). Marco and colleagues conducted a study on the utilization of r-tPA for treating ischemic stroke patients in Austria from 2006 to 2018. The findings indicate a rise in the use of r-tPA from 9.9% in 2006 to 21.8% in 2018 among stroke patients [12]. This trend is expected to continue as a result of the widening of the time window and further enhancements in the organization of stroke units. Nevertheless, a significant number of stroke patients still cannot receive thrombolysis because of time constraints and contraindications.

There are two main limiting factors for the wide clinical use of mechanical thrombectomy. To begin with, only around 10% of acute stroke patients have a proximal major artery occlusion that appears within 6 h of symptom onset [13]. An additional 9% of qualifying patients for mechanical thrombectomy are presented within 6 to 24 h of symptom start [14]. Second, not every hospital now has the necessary skills and resources for mechanical thrombectomy [15]. As a result, regional stroke units collaborate with supra-regional stroke units to ensure mechanical thrombectomy is available if indicated. In this scenario, patients are transferred to a supra-regional stroke unit after experiencing thrombolysis. This is dubbed as “drip and ship”. Obviously, this method significantly increases the onset-to-needle timings.

In conclusion, even if contemporary therapies are more accessible and effective to stroke patients by improving processes such as decreasing onset-to-door, door-to-needle, and onset-to-needle periods, they still have inherent limits. Only a small subgroup of stroke patients is eligible for these treatment paradigms underlining the great need for novel neuroprotective strategies to widen the therapeutic time window.

## 4. Neuroprotective Strategies and Approaches

The following chapters provide an overview of current strategies and concepts of neuroprotection for ischemic stroke.

### 4.1. Neuroprotectants

In the last decade, numerous agents showed promising neuroprotective potential in preclinical studies. However, none of it succeeded in translation into clinical practice [3]. Some of these promising candidates with completed or ongoing clinical trials will be discussed herein and are summarized in Table 1. An overview of ongoing clinical trials using “classical” neuroprotectants is additionally provided in Table 2.

NA-1 (nerinetide) is a 20 amino acid peptide that prevents the postsynaptic density protein 95 (PDS-95) from attaching to the NMDA receptor (NMDAR) subunit. It is suggested that the interaction inhibits neurotoxic downstream effects of NMDAR activation, including an excessive Ca^2+^ influx and nitric oxide generation [16] (see also section pathophysiology of stroke). Several preclinical investigations have shown the neuroprotective potential of this pathway, as demonstrated by reduced infarct size and improved neurological outcomes [17]. The preclinical evidence led to a multicenter randomized clinical trial including over 1000 participants using both NA-1 as a potential neuroprotective agent and recanalization therapy (ESCAPE-NA1) [18]. Unfortunately, the administration of NA-1 did not improve the neurological outcome in treated patients compared to the placebo group. However, the authors hypothesized that a drug-drug interaction of alteplase and NA-1 led to a decreased plasma level of NA-1 [19]. Finally, a subsequent study investigating NA-1 treatment in patients without thrombolysis treatment is on its way (ESCAPE-NEXT; NCT04462536). Additionally, a second study analyzing the prehospital administration of nerinetide in patients with suspected stroke is also ongoing (NCT02315443).

Sovateltide is an agonist of the g-protein coupled endothelin B receptors (ETBR). Selective stimulation of the ETBR significantly improves neurological and motor functions in stroke rats [20]. As a result, sovateltide administration after 4, 6, and 8 h after a stroke improves neurological prognosis. Interestingly, improved neural progenitor cell (NPC) differentiation, as well as improved mitochondrial shape and biogenesis, were identified in these stroke brains [21,22]. The preclinical evidence resulted in phase III clinical trial with 40 patients with acute ischemic stroke receiving sovateltide within 24 h after stroke onset [23]. Indeed, the authors were able to demonstrate improved neurological outcomes in patients with acute cerebral ischemic stroke as measured by improvements in the National Institute of Health Stroke Scale (NIHSS), modified Rankin Scale, and Barthel Index at 90 days post-treatment [23]. Taken together, Sovateltide is a promising neuroprotective candidate, asking for larger-sized studies in order to corroborate these findings.

Activated protein C (APC) is a protease with systemic anticoagulant, antiapoptotic effects, and anti-inflammatory effects. In preclinical research, APC was demonstrated to exert neuroprotection 4 h after stroke onset [24]. Due to its pleiotropic effects, a recombinant APC with a different amino acid sequence, 3K3A-APC, was created. Further, 3K3A-APC retains the cytoprotective properties of wild-type APC while greatly lowering its anticoagulant properties [25]. In a clinical phase I trial, 3K3A-APC was safe and well-tolerable in healthy adult volunteers [26]. A phase II clinical trial including 110 participants treated intravenously with 3K3A-APC combined with thrombectomy, thrombolysis, or both showed a trend toward lower hemorrhage rate (RHAPSODY; NCT02222714) [27]. A phase III trial with an estimated enrollment of 1400 participants is currently ongoing (NCT05484154; RHAPSODY-2).

Human urinary kallidinogenase (HUK) is a glycoprotein that regulates the kallikrein–kinin system and has shown promising results in stroke patients in several clinical trials. Wu and colleagues conducted a systematic review and meta-analysis on 18 studies with a total of 2676 participants [28]. The authors concluded that HUK combined with rt-PA significantly improved neurological recovery and the quality of life in stroke patients. However, the authors outlined several limitations of the studies, including a small sample size and methodological weaknesses. Only two studies documented the mortality during the follow-up period, implying that drug safety should be studied further, according to the authors [28].

Minocycline is an antibiotic drug from the group of tetracyclines and has shown to be a potent inhibitor of microglia activation, thereby suppressing the production of anti-inflammatory cytokines and mediators, including TNF-α, IL-1β, COX-2, and inducible nitric oxide synthase [29,30]. Administration of minocycline showed robust neuroprotective effects in preclinical stroke models. Naderie and colleagues systematically reviewed 35 preclinical studies; of those, 15 publications reported improved motor dysfunction and neurological deficits in models of focal cerebral ischemia. In nine studies, attenuated cognitive impairments and neurobehavioral dysfunctions were observed after minocycline treatment [31]. The preclinical evidence resulted in several clinical trials. A systematic review and meta-analysis including seven randomized controlled clinical trials (RCT) plus additional unpublished data suggest minocycline is a promising neuroprotective agent [32]. The neuroprotective effect was indicated by improvement in 3-month functional independence, Barthel index, and NIHSS score. However, several of the included RCTs have small patient numbers and are underpowered to detect differences in clinical endpoints, which may lead to bias in the meta-analysis. In addition, the combination of minocycline with magnesium was tested in clinical trials, although a systematic review stated no clinical benefit from magnesium sulfate infusion in overall stroke patients [33]. Nevertheless, the subgroup of patients with lacunar stroke that supplemented their meals with magnesium salt had improved functional outcomes [33]. Additional clinical trials employing minocycline alone and in combination with magnesium are now being conducted to expand the clinical evidence (see Table 2).

2-Hydroxy-5-(2,3,5,6-tetrafluoro-4-trifluoromethyl-benzylamino)-benzoic acid (Neu2000), also known as Nelonemdaz, is a multi-target neuroprotectant that aims to prevent damage from the NMDA receptor and free radicals. In two rodent stroke models, Neu2000 was shown to have neuroprotective potential by reducing infarct size and improving behavior, according to a study by Gwag and colleagues [34]. A phase II clinical trial involving 208 stroke patients who underwent endovascular reperfusion was conducted to investigate the benefits of Neu2000 administration [35]. The results showed a trend towards better scores on the modified Rankin Scale 12 weeks after stroke onset, but no significant difference. A phase III clinical trial is currently underway to clarify the efficacy of Neu2000 in hyperacute ischemic stroke and endovascular thrombectomy patients [36] (see Table 2).

Uric acid (UA) is a byproduct of purine metabolism and acts as an endogenous antioxidant. It can increase in response to oxidative stress situations such as stroke [37]. In several studies on acute ischemic stroke, higher UA levels have been linked to improved functional recovery due to its synergistic effects with alteplase [37]. For instance, Romanos and colleagues found that administering UA soon after a thromboembolic stroke can result in a smaller infarct volume and improved neurological function [38]. Furthermore, UA also increases the protective effects of rt-PA in the same thromboembolic rat study. A clinical phase IIb/III study involving 411 stroke patients evaluated the outcomes of those who received both UA and thrombolysis therapy [39]. The results showed that the therapy did not lead to a higher proportion of patients with excellent outcomes at 90 days post-stroke. However, the secondary analysis revealed that there was an improvement in early ischemic worsening, defined as an increase of ≥4 points in the NIHSS score within 72 h of treatment, as long as there was no hemorrhage or recurrent stroke [40]. Further clinical trials are necessary to determine the potential benefits of UA.

Toll-like receptor 4 (TLR4) is a member of the pattern recognition receptors and is involved in activating the innate immune response and in triggering the inflammatory response during the pathophysiology of various diseases, such as ischemic conditions [41]. This has led to high interest in the use of substances such as ApTOLL, a potent TLR4 antagonist, to help alleviate these conditions. Studies on rodents have shown that ApTOLL has neuroprotective effects, such as reduced infarct volume and improved neurobehavioral outcomes in stroke models [42]. The first study in humans has confirmed the safety of ApTOLL [43]. A clinical phase Ib/IIa study, which was estimated to enroll 151 patients with confirmed large vessel occlusion who received reperfusion therapy, was completed in 2022. However, the official results of the study have not yet been released (NCT04734548).

Edaravone is a free radical scavenger that affects various signaling pathways that regulate apoptosis, microglia activation, and long-term neuroinflammation in addition to its antioxidant activity [44]. In preclinical studies, Edaravone showed neuroprotective effects such as BBB stabilization, reduction of brain edema, and improved neurological outcomes [44]. The emerging preclinical evidence led to numerous clinical trials and even to approval for clinical use in Japan. Fidalgo and colleagues recently performed a meta-analysis including 19 randomized controlled trials and observational studies [45]. The analysis showed that Edaravone treatment resulted in improved neurological outcomes and decreased mortality. However, the authors noted that the majority of studies were performed in Asia, especially Japan, and studies including patient populations outside of Asia are required to confirm the neuroprotective potential. Further, Edaravone combined with Dexborneol may result in a synergistic antioxidant and anti-inflammatory effect. Indeed, a recently completed clinical research including over 1000 stroke patients revealed that the combination of Edaravone with Dexborneol outperformed the benefits of Edaravone alone in terms of outcome [46]. Currently, there are two more studies testing Edaravone alone and in comparison to the combination of Edaravone and Dexborneol (see Table 2).

### 4.2. Stem Cells and EVs

Cumulative evidence suggests that stem-cell-based therapy in different neurological and immune diseases may be beneficial. The goal of stem-cell-based therapy in ischemic stroke is both (I) to immediate neuroprotection and (II) to initiate subacute and long-term neuro-regeneration. In the following report, current findings and mechanisms of stem-cell-based stroke therapy will be discussed. Stem cells intended for stroke therapy could be categorized into three main cell types, including NPC, bone marrow stem cells (BMSC), and mesenchymal stem cells (MSC) [47]. An overview of ongoing and completed clinical trials investigating stem-cell-based therapy in stroke patients is provided in Table 3 and Table 4, based on data obtained from the ClinicalTrials.gov website.

Fukunaga and colleagues demonstrated that mice implanted with rat-derived NPC and mesenchymal tissue showed enhanced cognitive function after stroke [48]. As a result, the researchers showed that the implanted tissue developed into mature CNS tissue, including neuron-like cells and the development of new vascular networks. Human NPC (hNPC) generated from a human fetus forebrain and grown in vitro received interest in the following years. Transplanted hNPC were observed to develop into neurons, oligodendrocytes, and even astrocytes in the brains of stroke rats [49]. Importantly, with the help of the electrophysiological recording and immune-electron microscopy, it has been shown that the implanted hNPC was connected to the striatal host cells after differentiation [49]. This lends credence to the hypothesis that implanted NPC can, in fact, restore missing brain tissue. As the next step for a bench-to-bedside translation, Pollok and colleagues generated an hNPC line under good manufacturing practice (GMP) named CTX0E03 [50]. CTX0E03 implanted into the putamen in stroke rats revealed enhanced behavioral recovery and improved endogenous neurogenesis in a dose-dependent fashion [51]. Interestingly, Smith and colleagues proved that the success of CTX0E03 therapy is thus dependent on the implantation site and stroke lesion topology [52]. This promising preclinical data resulted in a phase I trial. In a first-in-man study, thirteen participants received a single stereotactic implantation located in the ipsilateral putamen with a specific dosage between 2 and 20 million CTX0E03 cells 6–60 months after ischemic stroke [53]. Indeed, the CTX0E03 treatment was associated with better neurological function, indicated by improvements in NIHSS, Ashworth scale, and Barthel Index scores [53]. Importantly, no adverse effects were observed. This was followed by the phase II study (PICES II), which again showed improvements in neurological functions, indicated by enhanced upper limb function (Table 3) [54]. The follow-up study PICES III is currently ongoing (NCT03629275).

BMSCs, like NPC, have been proven to be capable of differentiating into cells with neural characteristics [55]. BMSCs were extensively studied in preclinical stroke models. Chena and colleagues, for the first time, demonstrated that intracerebral implanted BMSC migrate to the lesion site and differentiate into phenotypic neural cells in ischemic rats [56]. Moreover, significant recovery of somatosensory behavior and improved neurological severity scores were found in animals implanted with BMSC. The migratory potential was further studied in terms of intravenous administered BMSC in stroke rats [57]. Here, the authors observed reduced infarct lesion size and better functional outcomes in animals receiving BMSC injection up to 72 h after stroke induction. This together led to several clinical trials. However, Prasad and colleagues revealed in the first multicenter randomized controlled trial intravenous given BMCSs are safe but have no beneficial effect on stroke outcome (Table 3) [58].

Wislet-Gendebien and colleagues, for the first time, confirmed that MSCs could differentiate in excitable neuron-like cells [59]. In addition, MSCs have the potential to differentiate into glial cells and endothelial cells and are able to migrate into the infarct region after peripheral injection in ischemic stroke models [60,61]. MSC can be obtained from different sources of tissue, such as umbilical cord stromal cells, umbilical cord blood (UB), adipose-derived stromal cells (ADSCs), and even dental tissues [62,63,64]. Several preclinical stroke-related studies using MSCs were conducted. The meta-analysis of Vu and colleagues evaluated 46 preclinical studies using MSC treatment for ischemic stroke, whereas 44 of these showed significantly improved outcomes [65]. As a result, the trials revealed an overall very substantial treatment effect and very robust results in terms of different species, delivery routes, MSC sources, time, dose, and the presence of comorbidities [65]. MSCs have been demonstrated to alter various elements of stroke pathogenesis, including BBB stabilization, brain edema reduction, and neuroinflammation [66]. Regarding the latter, MSC treatment lead to decreased microglia activation, immune cell migration, and diminished levels of pro-inflammatory cytokines Interleukin (IL)-1α, IL-1β, IL-6, and tumor necrosis factor α (TNF-α) among with elevated levels of anti-inflammatory cytokines IL-4, IL-10, interferon-β [66]. This preclinical evidence led to clinical trials (Table 3 and Table 4). Indeed, Jaillard and colleagues’ clinical phase II investigation employing autologous MSC administered intravenously indicated better motor recovery via sensorimotor neuroplasticity [67]. However, a Phase III clinical trial with MSC given within 90 days after stroke onset detected no improved outcome in stroke patients [68]. Several clinical trials using mainly umbilical-cord-derived MSC are ongoing (Table 3).

Apart from applying stem cell therapy directly, using extracellular vesicles (EV) secreted by stem cells gained even more interest in the past years. EVs consist of a lipid bilayer structure with a diameter between 30 and 1000 nm. The biological function of EVs is determined by their specific content, such as DNAs, RNAs, and proteins [69]. Importantly, compared to stem cells, EVs are easier to obtain, have an inferior risk of malignant transformation, and have fewer ethical issues. Several preclinical studies demonstrated the neuroprotective and neuroregenerative potential of EVs in various stroke models. For example, intravenous application of MSC-derived EVs led to improved neurological impairment and long-term neuroprotection associated with elevated angiogenesis in stroke mice [70]. Mechanistically, modulation of the immune system indicated by reversed postischemic lymphopenia and modulated T-cell levels in the peripheral blood were suggested. Xia and colleagues showed that the application of MSC-derived EVs in stroke rats decreased the infarct volume and the autophagy activity in the ischemic tissue [71]. Numerous additional studies underlined the neuroprotective potential of EVs in the preclinical setting [72]. Of notice, these effects were also detected by EVs produced from other stem cell lines, such as NPC as well as MSC-derived EVs. However, the exact mechanism of the observed effects remains elusive [73]. More recent studies suggest a prominent role of miR-content within the EVs in stroke settings. For example, miR-21a, miR-26a, and miR-126 have been described as pivotal players [74,75,76]. Recent studies suggest that the EV content could be modulated due to specific environments. For example, EVs derived from MSC incubated in lithium have been more effective than native EVs [77]. Furthermore, Alehossein and colleagues considered the possibility of transplanting EVs produced from multi-parental cells of physically active persons [78]. Adapting the EV material to its unique use may be a critical aspect of clinical EV application. Despite the growing body of preclinical evidence, no translational clinical trial of EV-based stroke treatments has been completed yet. However, a research trial is currently underway to investigate EVs as a potential biomarker for the profiling of stroke patients in order to personalize rehabilitation therapies (NCT05370105).

### 4.3. Microbiota–Gut–Brain Axis

The gastrointestinal tract is a major immune organ with the largest reservoir of immune cells, representing around 70% of the entire immune system [79]. The bidirectional communication between the central and the enteric nervous system is known as the microbiota–gut–brain axis (MGBA) [80]. An increasing number of studies indicate a critical role of the MGBA in systemic immune response following neurological diseases [81].

Cumulating evidence suggests that the MGBA is implicated in stroke pathophysiology and has a major impact on the outcome. Importantly, around 50% of stroke patients develop gastrointestinal complications such as dysmotility, gut hemorrhage, constipation, gut incontinence, and even gut-origin sepsis [82]. In addition, gut microbiota dysbiosis has been shown to contribute to neurobehavioral deficits, affecting the neuroinflammatory response and finally worsening stroke outcomes [83,84]. Even if the role of the MGBA with respect to stroke is not fully understood, recent studies give insights into possible mechanisms.

Benakis and colleagues demonstrated for the first time that antibiotic-induced alterations of the gut microbiota affect the stroke outcome positively by increasing intestinal regulatory T cells and decreasing γδ T cells. This dysbiosis reduces the migration of effector T cells from the gut to the leptomeninges after stroke, resulting in decreased infarct size and increased behavioral outcomes in stroke mice [85]. Moreover, Xu and colleagues reported that stroke itself induces gut dysbiosis with enterobacteriaceae overgrowth by inducing intestinal ischemia and enhanced nitrate production. This, in turn, expands the infarct volume [86]. In stroke mice, however, providing superoxide dismutase or aminoguanidine to reduce nitrate concentration leads to decreased expansion of enterobacteriaceae species, low levels of systemic inflammation, and diminished infarct volume [86]. These findings underscore the importance of the gut microbiome in terms of stroke outcomes. Following that, Xia and colleagues developed a gut dysbiosis index (SDI) based on specific gut microbial dysbiosis patterns, examining whether or not these patterns were associated with brain injury and early outcomes [87]. They were able to demonstrate that the SDI positively correlates with the NIHSS in stroke patients. Furthermore, in a rat stroke model in which mice received fecal transplants from high-SDI patients and transplants from low-SDI patients, this link was causally associated. Interestingly the proportion of IL-17^+^ γδ T cells in the gut was identified as a major aspect of outcomes related to gut microbiome dysbiosis. Wang and colleagues recently explored the effect of the gut microbiota in a type 2 diabetes (T2D) mouse model, a comorbidity known to affect stroke outcome [88]. In fact, the proportion of butyrate-producing bacteria and butyrate in the gut microbiome is decreased in T2D patients and is associated with attenuated brain injury. The authors were able to demonstrate that transplanting fecal samples from mice treated with sodium butyrate or a control material could reduce T2D-associated stroke outcome deterioration [88]. Hence, specific targeting of the gut–brain relationship may prove to be promising, too, for future neuroprotective therapies.

## 5. Conclusions and Perspective

Current treatment strategies for ischemic stroke are limited to causal vessel recanalization, from which only a minority of stroke patients benefit. Despite enormous efforts in neuroprotective research, there has yet to be a clinical translation of an adequate neuroprotective strategy. Nevertheless, several promising neuroprotective approaches are available. Neuroprotective agents, including NA-1, Sovateltide, 3K3A-APC, HUK, minocycline, and Edaravone, showed robust results in preclinical stroke settings and are already transferred to clinical trials. Further, stem-cell-based neuroprotective therapies have promising aspects, and several clinical trials, especially with UC-MSC, are ongoing. EVs derived from stem cells show distinct neuroprotective effects in preclinical stroke settings. Furthermore, preconditioning EVs for specific application purposes may be an important part of future clinical EV applications. However, a transfer of EVs to a clinical trial is missing until today. Emerging neuroprotective notions, such as addressing the microbiota–gut–brain axis, open up new avenues of research in the field of neuroprotection. Future trials will elucidate if one neuroprotectant or a combined strategy with different neuroprotective targets may help widen the therapeutic window for stroke patients.

## Figures and Tables

**Table 1:** Promising neuroprotective Candidates. Abbreviations: APC: Activated Protein C; ETBR: Endothelin B receptor; HUK: Human urinary kallidinogenase; NMDAR: N-methyl-D-aspartate receptor; NPC: Neurogenic Progenitor Cell; PDS-95: Postsynaptic density protein 95; RCT: Randomized Clinical Trial; rt-PA: Recombinant Tissue Plasminogen Activator; UA: Uric Acid.

Substance	Mechanism	Evidence	Limitations	Perspective
NA-1	- Prevent PDS-95 from attaching to NMDAR, thereby inhibiting excessive Ca^2+^ influx and generation of nitric oxide.	- Reduced infarct size and improved neurological outcome have been observed in preclinical stroke models.	- An RCT (ESCAPE-NA1) showed that the neurological outcome did not improve. - There is a hypothesis that the drug-drug interaction of alteplase and NA-1 led to a decreased plasma level of NA-1.	- An ongoing RCT is investigating NA-1 treatment in patients without thrombolysis treatment (ESCAPE-NEXT).
Sovateltide	- Agonist of ETBR. - Improve NPC differentiation and improve mitochondrial shape as well as biogenesis, in ischemic brains.	- A Phase III RCT revealed improved neurological outcome 90 days post-treatment (NCT04046484).	- RCT only involved a sample size of 40 participants.	- A larger-sized randomized controlled trial RCT is needed to confirm the findings.
3K3A-APC	- Recombinant APC with lower anticoagulant properties has antiapoptotic and anti-inflammatory effects.	- A Phase II RCT showed that patients treated intravenously with 3K3A-APC combined with thrombectomy, thrombolysis, or both showed a trend towards a lower hemorrhage rate (RHAPSODY).	- The RCT only included a small sample size of 110 participants.	- A Phase III trial with an estimated enrollment of 1400 participants is currently ongoing (RHAPSODY-2).
HUK	- Regulates the kallikrein–kinin system.	- Several RCT has been conducted. - A meta-analysis of 18 RCTs revealed that HUK combined with rt-PA significantly improved the neurological recovery and quality of life in stroke patients.	- Small sample size and methodological weaknesses in the studies. - Only two studies documented the mortality rate during the follow-up period.	- More studies, especially those documenting the mortality rate, are necessary to investigate the drug’s safety.
Minocycline	- Antibiotic drug from the group of tetracyclines. - Has been shown to be a potent inhibitor of microglia activation, thereby suppressing the production of anti-inflammatory cytokines and mediators.	- Several RCTs revealed a neuroprotective effect, indicated by improvement in 3-month functional independence, Barthel index, and NIHSS score.	- several RCTs had small sample sizes and were underpowered. - The combination with magnesium showed no clear results.	- Additional clinical trials employing minocycline alone and in combination with magnesium are now being conducted to expand the clinical evidence (NCT05512910; NCT05032781).
Neu2000	- Aims to prevent damage mediated by the NMDA receptor and free radicals.	- A phase II RCT involving 208 stroke patients who underwent endovascular reperfusion was conducted. - The results showed a trend towards better scores on the modified Rankin Scale 12 weeks after stroke onset, but there was no significant difference.	- No significant neuroprotection has been found in RCTs to date.	- A phase III clinical trial is currently underway to clarify the efficacy in hyperacute ischemic stroke and endovascular thrombectomy patients (RODIN).
Uric Acid	- Byproduct of purine metabolism and acts as an endogenous antioxidant.	- A clinical phase IIb/III study involving 411 stroke patients showed that the therapy did not result in a higher proportion of patients with excellent outcomes at 90 days post-stroke. - A secondary analysis revealed that there was an improvement in early ischemic worsening.	- There was no significant difference in the primary endpoint of the RCT (NCT00860366).	- Further clinical trials are necessary to determine the potential benefits.
ApoTOLL	- TLR4 antagonist. - TLR4 plays a role in activating the innate immune response and triggering the inflammatory response.	- Preclinic studies have shown neuroprotective effects. - A Phase I RCT demonstrated safety.	- No official results from a Phase II RCT have been released till date.	- A Phase Ib/IIa RCT was completed in 2022, but the official results of the study have not yet been made public (NCT04734548).
Edaravone	- Free radical scavenger that has been shown to regulate apoptosis, microglia activation, and long-term neuroinflammation, as well as exhibiting antioxidant activity.	- A meta-analysis of 19 RCTs revealed improved neurological outcomes and decreased mortality. - The combination of Edaravone and Dexborneol was found to be even more effective than Edaravone alone in terms of outcome.	- The majority of studies on Edaravone were conducted in Asia, particularly in Japan.	- Further studies are underway to test the efficacy of Edaravone alone and in combination with Dexborneol (NCT05024526; NCT05032781).

**Table 2:** Ongoing clinical trials investigating neuroprotective agents in ischemic stroke patients.

NCT Number; Name	Drug	Phase	Route	Estimated Enrollment	Country
NCT04462536 (ESCAPE-NEXT)	NA-1 (nerinetide)	III	intravenous	1020	Canada
NCT02315443 (FRONTIER)	NA-1 (nerinetide)	III	intravenous	586	Canada
NCT05484154 (RHAPSODY-2)	3K3A-APC	III	intravenous	1400	United States
NCT03320018 (H2M)	Hydrogen and Minocycline	II and III	intravenous and oral	100	United States
NCT03347786	Verapamil	I	intravenous	20	United States
NCT05032781	Minocycline and magnesium	I	intra-arterially	24	United States
NCT05041010 (RODIN)	Neu2000	III	intravenous	496	Korea
NCT05512910 (MIST-B)	Minocycline	IV	oral	90	China
NCT05124353	Cerebrolysin	II	intravenous	100	Poland
NCT05024526	Edaravone	N/A	intravenous	80	China
NCT05249920 (TASTE-2)	Edaravone Dexborneol	III	intravenous	1362	China

**Table 3:** Overview of ongoing (recruiting) clinical trials investigating stem-cell-based therapies in ischemic stroke patients. Abbreviations: N/A: not available; UC-MSC = umbilical cord-derived mesenchymal stem cells.

NCT Number	Cell Line	Phase	Administration	Estimated Enrollment	Treatment after Stroke Onset	Country
NCT04811651	UC-MSC	II	Intravenous	200	within 6 months	China
NCT05292625	UC-MSC	I	Intravenous	48	72 h and 3 months	Vietnam
	UC-MSC	II	intrathecal		72 h and 3 months	
NCT04280003	Alogenic adipose tissue-derived MSC	II	Intravenous	30	within 4 days	Spain
NCT04097652	UC-MSC (UMC119-06)	I	Intravenous	9	48 to 168 h	Taiwan
NCT04434768	UC-MSC (UMSC01)	I	intraarterial and intravenous	14	N/A	Taiwan
NCT04093336	UC-MSC	I and II	Intravenous	120	within 7 days	China

**Table 4:** Overview of completed clinical trials using stem-cell-based therapies for ischemic stroke treatment. Abbreviations: BMSC: bone-marrow-derived mesenchymal stem; EPC: endothelial progenitor cells; hNPC: human neural progenitor cells; UCB: umbilical cord blood.

NCT Number; Name	Cell Line	Phase	Route	Participants	Time Point	Result
NCT01678534 (AMASCIS-01)	allogeneic stem cells from adipose tissue	II	intravenous	19	Within 2 weeks	No end points were statistically significant.
NCT01501773 (InVeST)	autologous bone marrow mononuclear cells	II	intravenous	120	median of 18.5 days	No beneficial effect on stroke outcome.
NCT02117635 (PISCES-II)	hNPC (CTX0E03)	II	intracerebral	23	2–13 months	Improvements in upper limb functions.
NCT01468064 (AMETIS)	EPCs	II	intracerebral	20	7 days	No significant difference in neurological or functional improvement, but fewer serious adverse events.
NCT00875654 (ISIS)	MSC	II	intravenous	31	Within 6 weeks	Improved motor recovery.
NCT03004976 (CoBIS 2)	UCB stem cells	II	intravenous	79	3–10 days	Not yet published
NCT01436487 (MASTERS)	multipotent adult progenitor cells	II	intravenous	134	1–2 days	No beneficial effect on stroke outcome.
NCT01716481 (STARTING-2)	MSC	III	intravenous	54	90 days	No improved outcome

## Data Availability

Data sharing not applicable. No new data were created or analyzed in this study. Data sharing is not applicable to this article.
